# Diverse school community engagement with the North Carolina active routes to school project: a diffusion study

**DOI:** 10.1186/s12966-019-0889-z

**Published:** 2019-11-29

**Authors:** Seth LaJeunesse, Sam Thompson, Nancy Pullen-Seufert, Mary Bea Kolbe, Stephen Heiny, Cathy Thomas, Edward R. Johnson

**Affiliations:** 1 Highway Safety Research Center, University of North Carolina at Chapel Hill, Chapel Hill, NC USA; 2 Division of Public Health, North Carolina Department of Health and Human Services, Raleigh, NC USA; 3 Division of Bicycle and Pedestrian Transportation, North Carolina Department of Transportation, Raleigh, NC USA

**Keywords:** Physical activity, Diffusion of innovations, Safe routes to school, School health, North Carolina

## Abstract

**Background:**

Schools located in rural parts of the United States and North Carolina have benefited proportionally less from the federal Safe Routes to School (SRTS) program than their more urban counterparts. We investigated whether and how diverse elementary and middle school communities throughout North Carolina have engaged in a SRTS-inspired, multi-sectoral initiative called the Active Routes to School (ARTS) project over the course of 5 years (2013 through 2017).

**Methods:**

Analyses included a study sample of 2602 elementary and middle schools in North Carolina, 853 that participated in the ARTS project over the five-year study period and 1749 that had not. Statistical models controlling for county- and school-level confounders predicted schools’ involvement in walking and bicycling-promotive events, programs, and policies over time.

**Results:**

Schools’ engagement with ARTS Project programming increased significantly over the study period, with 33% of eligible schools participating with the project by the end of 2017. Participation was most common in promotional events. Such event participation predicted engagement with regularly recurring programming and school- and district-level establishment of biking- and walking-facilitative policies. Lower income schools were more likely to establish recurring bike and walk programs than wealthier schools, whereas rural schools were less likely than city schools to participate in promotional events, yet equally as likely as other schools to participate in recurring bike and walk programs.

**Conclusions:**

Schools’ engagement with the North Carolina ARTS Project diffused despite many schools’ rural geographies and lower socioeconomic status. Further, participation in one-time promotional events can portend schools’ establishment of recurring walking and biking programs and supportive policies.

## Background and introduction

In 2005 with the passage of the federal transportation bill, Safe, Accountable, Flexible, Efficient Transportation Equity Act: A Legacy for Users (SAFETEA-LU), the United States federal government established dedicated funding for a Safe Routes to School (SRTS) program to benefit k-8th grade school children. Section 1404(b) of this legislation described the purposes of the program: (1) “enable and encourage children, including those with disabilities, to walk and bicycle to school; (2) to make bicycling and walking to school a safer and more appealing transportation alternative, thereby encouraging a healthy and active lifestyle from an early age; and (3) to facilitate the planning, development, and implementation of projects and activities that will improve safety and reduce traffic, fuel consumption, and air pollution in the vicinity of schools.” [1].

Under this legislation, state Departments of Transportation (DOTs) were required to establish a full-time State SRTS Coordinator position. These Coordinators were to develop calls for funding proposals and prioritize the distribution of federal funding to improve bicycling and walking conditions for children traveling to and from schools. Unique among federal transportation programs, the SRTS program reimbursed funding recipients 100% of the cost of implementing the projects—all other federal transportation programs during this time required 20% state or local matches [2]. These projects were intended to support walking and bicycling to and from school by incorporating—directly or indirectly—five project components, most often referred to as the “five E’s” of transportation safety: engineering, education, encouragement, enforcement, and evaluation [1].

More than a decade’s worth of research and evaluation suggests that SRTS represents a promising suite of strategies to enhance physical activity among school populations. McDonald and colleagues [3] found that schools that implemented comprehensive safe routes to school programs—initiatives that included educating children about safe biking and walking practices, encouraging use of these modes, and making built environment changes for safe biking and walking to school—demonstrated a 31% increase in biking and walking to school over a five-year period. Similarly, in a study involving 53 schools in five U.S. states, Stewart, Moudon, and Claybrooke observed significant increases in biking and walking to school after the schools implemented infrastructure (e.g., sidewalks, bike lanes) and non-infrastructure (e.g., promotional events, enhanced school zone school speed enforcement) projects [4].

Theoretical and empirical studies support the philosophy underlying SRTS. In a review of intervention studies designed to increase active school travel, Larouche, Mammen, Rowe, and Faulkner concluded that more comprehensive interventions—i.e., those that complement educational and promotional activities with changes to the built environment—seem to result in greater increases in active school travel [5]. Further, they observed that studies that featured interventions with longer follow-up periods were likely to achieve greater modal shifts. Such findings are in keeping with social-ecological models of human behavior, wherein interventions that operate across multiple levels—individual, interpersonal [6], and community are most likely to produce lasting results [7, 8].

Safe Routes to School programs also appear to be associated with increased child bicyclist and pedestrian safety in the U.S. Examining traffic crash records involving school age children in 18 U.S. states, Dimaggio, Frangos, and Li observed significant reductions in bicycle and pedestrian injury and fatalities for this age group relative to adult populations, suggesting that the federal safe routes to school program played a role in these safety gains [9].

Nonetheless, rural communities appear to have benefited less from safe routes to school programming. This disparity may exist for many reasons. For one, rural areas have tended to receive less SRTS funding than their more urban counterparts, partly as a result of requesting proportionately less funding [10]. Moreover, stakeholders in rural communities often find it difficult to develop grant proposals to support active living, including SRTS [11]. Residents of rural areas also tend to have lower access to health information from sources including primary care providers and specialists compared to their urban counterparts [12].

In North Carolina’s predominantly rural Appalachian region, researchers found that basic pedestrian and cyclist safety features (e.g., sidewalks, crosswalks, shoulders) that experts consider critical for supporting active living among residents [13] were often missing or in states of disrepair [14]. Additional factors likely contribute to the rural-urban health divide, including, but not limited to transportation challenges due to greater distances among destinations, lower population densities, limitations regarding health information and communication, and a lack of recreational and other physical activity promoting infrastructure close to rural residents’ homes, schools, or workplaces [15, 16].

And though health-promotive innovations can effectively diffuse through a population, such diffusion may be too limited or unfold too slowly to improve the health and well-being of those living in more remote places farther from corridors of political power [17]. This is problematic, as recent work suggests that residents of rural areas tend to engage in less leisure-time physical activity [18] and are more likely to be obese [19, 20].

### Partnership overview

To positively impact school communities across North Carolina (NC) and expand the reach of Safe routes to School to more rural regions of the state, program leaders with the state’s Department of Transportation (DOT) and Division of Public Health (DPH)—established a collaborative partnership project called Active Routes to School (ARTS). Through this partnership, NCDOT allocated federal SAFETEA-LU transportation funding to support the work of a designated DPH staff person who served as a Project Manager and 10 Active Routes to School Regional Coordinators (ARTS Coordinators), each stationed in one of 10 “lead health agencies” around NC. The ARTS Coordinators were responsible for engaging school districts, schools, and their partners in a region with 10 counties on average, thereby serving all 100 counties in NC.

The ARTS Coordinators worked with the NCDOT SRTS Program Manager, the NCDPH Project Manager, as well as technical consultants with the University of North Carolina Highway Safety Research Center (HSRC), to develop yearly work plans and implement interventions that together comprised the ARTS Project. In the yearly work plans, the ARTS Coordinators described their goals for engaging in several key processes. These processes included meetings with municipal partners, school district and school administrators and staff, educational programming (e.g., demonstrating the Let’s Go NC! school-based bicycle and pedestrian safety curriculum), promotional events, recurring bicycling and walking safety and promotional programs, and active school travel-facilitative policies. They also served as brokers between school communities and state and local transportation planners and engineers who could design changes to the built environment. It is worth noting that the NCDPH and ARTS Coordinators were tasked with implementing the “non-infrastructure” elements of the federal Safe Routes to School Program (e.g., education and encouragement activities, programs, and policies) rather than on working with schools to install bike- and walk-supportive infrastructure.

Though a few regional Metropolitan Planning Organizations (MPOs) and cities have formed arrangements with regional and local health departments, NC has been the only US state to establish an enduring partnership between the state’s DOT and DPH. In so doing, the ARTS Project has remained the only project to explicitly integrate the resources of the state Department of Transportation (DOT) with outreach and engagement-related strengths of the state and local public health sectors. Further, in most states, schools, districts, and municipalities apply to the state DOT for SRTS funding. In North Carolina and under the ARTS Project, the ARTS Coordinators proactively reached out to school communities to tailor programming to address locally determined needs using existing community resources.

By design, the ARTS Coordinators also aligned their practice with several of the principles of community engagement [21]. That is, by living in the regions in which they worked, ARTS Coordinators more readily drew upon their knowledge about the community’s culture, economic conditions, social and political networks, norms and values, etc. to co-develop context-appropriate programs. They also established relationships with community partners and built trust with formal and informal leaders to identify and help mobilize community assets to implement community health-related decisions [22].

In this study, we explore how over the course of 5 years, a diverse array of NC-based K-8th grade schools—paying particular attention to traditionally disadvantaged rural school communities—engaged with four ARTS interventions. Specifically, we sought answers to the following research questions.

- To what extent did traditionally disadvantaged rural school communities engage in the North Carolina ARTS Project from 2013 through 2017?; and
- How did the nature of participation in ARTS programming vary as a function of schools’ rural locations, income, academic achievement status, and racial/ethnic populations in North Carolina?

### Study sample

The study sample includes 2602 public and private schools offering Kindergarten through eighth grade enrollment in NC. These schools represented an estimated 98% of K-8 schools in the state as of the 2016–2017 school year. The 52 schools not included in the analysis involved those that lacked school demographic information or had closed at some point during the 2013–2017 study period.

Each month, the Active Routes to School Coordinators entered school-, county-, and region-level programming information into Formstack, an online form builder and data repository. This procedure allowed the research team to identify which schools participated in which programmatic activities and when they did so. The team identified “participating schools” as those schools that engaged in ARTS Project-promoted events, programs, or policies any time from 2013 through 2017 (*n* = 853). “Non-participating schools” constituted all other K-8th grade schools in NC that had not participated in ARTS programming any time from 2013 through 2017 (*n* = 1749). According to schools’ academic growth status, Census-defined locale, and racial/ethnic composition, participating schools were not significantly different than non-participating schools. However, elementary schools and schools located in counties that enrolled higher proportions of overweight or obese students were overrepresented among ARTS Project-participating schools. On the other hand, medium income schools were underrepresented among ARTS Project-participating schools (Table 1).

**Table 1:** Non-participating and participating schools’ composition (*N* = 2602)

	Non-participating schools (*n* = 1749)	Participating schools (*n* = 853)	
	%	%	*p*
County-Level			
*Childhood Weight Status*			0.000
Overweight/Obese	30.8	43.1	
*Rurality*			0.225
Rural	45.2	43.8	
Suburban	25.9	21.9	
Urban	28.9	34.2	
School-Level			
*Academic Growth Status*			0.090
Exceeded	27.6	26.2	
Met	47.8	54.1	
Not Met	24.6	19.9	
*Elementary School*			0.000
Yes	77.4	87.6	
*School Locale*			0.076
City	29.6	32.6	
Suburb	20.0	20.8	
Town	11.7	15.0	
Rural	38.6	31.5	
*School Income Level*			0.002
Low	16.2	19.6	
Medium	62.9	55.5	
High	20.9	24.8	
*School Racial/Ethnic Demographics*			0.952
Hispanic	14.1	14.8	
Black	23.8	25.1	
White	51.2	50.0	
Two Races	3.7	3.8	

*Note.* Presented *p* values reference results of likelihood ratio χ^2^ tests

### Dependent variables

In setting up the statistical analysis, the research team divided dependent *variables* into three inter-related categories: (1) promotional events; (2) recurring educational or promotional programs; and (3) bicycling- and walking- facilitative policies. Promotional events were further divided into participation in national Walk to School Day (WTSD) and Bike to School Day (BTSD) celebrations. As the names imply, WTSD and BTSD occur on a single day—i.e., each October and May, respectively—and are typically intended to demonstrate the benefits of walking and bicycling to school and safe travel behaviors.

Recurring educational or promotional programs (Programs) represent a class of activities that occur at least monthly. Such activities include: “walking school buses” and “bicycle trains”, whereby groups of students walk or bicycle together to school under adult supervision; “remote drop-off or pick-up” programs where instead of driving to the school student drop-off and pick-up occurs at an off-campus location and students walk, with adult supervision, the remainder of the trip to school; and “walk or bicycle at school” programs, including walking or bicycling activities that take place on the school campus.

Finally, biking- and walking- facilitative policies (Policies) comprise procedures that guide schools’ biking and walking activities and programs. These policies are instituted at the level of individuals schools or school districts. In cases where policies applied to districts, we indicated in the analysis that all K-8 schools in the district were impacted by these policies.

### Independent variables

#### County-level data

The independent variables we used in the analyses pertained to county-level and school level factors. County-level data included each county’s rural, suburban, and urban designations according to the North Carolina Center for Public Policy Research [23]. “Rural” counties are those with average population densities of 250 people or less per square mile; “Suburban” counties are those with an average population density between 250 and 750 people per square mile; and “Urban” are those with average populations exceeding 750 people per square mile.

Child overweight and obesity proportions data derived from the North Carolina Pediatric Nutrition and Epidemiology Surveillance System and pertain to children ages five to 11 by county [24]. Counties with overweight or obese child populations above 48% (one standard deviation above the mean), were categorized as “overweight/obese” counties.

#### School-level data

Data collected on individual schools derived from the U.S. Department of Education, National Center for Education Statistics (NCES). Yearly data gleaned from the NCES included schools’ elementary school status—a categorical variable indicating whether a school was designated an elementary school, or “schools beginning with grade 6 or below and having no grade higher than 8” [25]. NCES also provided data on schools’ income levels, a measure based upon the distribution of schools’ student population eligible to receive free or reduced-priced meals. Schools that enrolled fewer than 40% of student eligible to receive assistance from the National School Lunch Program, were categorized as “high-income” schools; those with between 75 and 40% of enrolled students eligible for the program were categorized as “medium-income” schools; and schools with greater than 75% of program eligible students were categorized as “low-income” schools [26]. We also used NCES data on the schools’ racial/ethnic demographics, i.e., the proportion of students at each school identifying as Black, White, Hispanic, or of two races. Lastly, the team included each schools’ U.S. Census-designated locale (i.e., city, suburb, town, rural) into the analysis. Schools’ locale signified a schools’ location in relation to an urbanized area. Size differentiates city and suburb designations and proximity to urbanized areas differentiates town and rural assignments.

Schools’ *Academic Growth Status*—the North Department of Public Instruction [27] provided a measure of schools’ “academic growth status”, which reflects schools’ yearly Education Value-Added Assessment System (EVAAS) academic growth scores. The “growth status” categories included “exceeded” (EVAAS growth scores of 85–100), “met” (EVAAS growth scores of 70–85), and “not met” (EVAAS growth scores below 70).

The calendar *Year* ARTS Coordinators provided the dates that each school of participated in various ARTS-promoted activities, i.e., 2013 (baseline) through 2017.

#### Data analysis

**Fig. 1:**
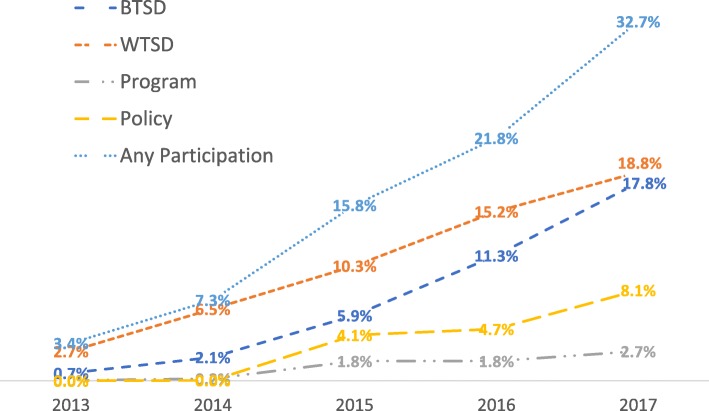
Cumulative school-level participation in ARTS Project outcomes of interest from 2013 through 2017 (*N* = 2602)

To assess the degree to which K-8 schools in NC participated in the ARTS project, the authors plotted schools’ cumulative participation in ARTS Project outcomes (i.e., BTSD, WTSD, Program, Policy, and a summation of all outcomes to create an “Any Participation” variable) from 2013 through 2017 (Fig. 1).

In addition to K-8th grade school population-level participation in the ARTS project, the research team was most interested in understanding which county- and school-level factors explained schools’ participation in ARTS programming, including Bike to School Day (BTSD) and Walk to School Day (WTSD) celebrations; at least monthly recurring promotional or educational programs (Program); and the institution of school district- or school-level policies designed to promote walking and bicycling as part of school procedure (Policy). A total of 13,010 unique observations (i.e., 2602 schools observed over the 5-year study period [2602 schools × 5 years = 13,010 observations]) were used in the analyses. About 20% of schools were missing data for some of the study years on the schools’ income—reported every other year for private schools in NC—and yearly academic growth status. These missing data were imputed using multiple imputation by chained equations [28].

We estimated two-level (i.e., schools nested within counties), mixed effects logit models using Stata version 15 [29] software for the four programmatic outcomes of interest (i.e., BTSD, WTSD, Program, and Policy). Initially, the team included county-level rural, suburban, and urban designations in the models, yet these variables failed to explain any of the outcomes and were therefore removed to produce more parsimonious models. In the final models and consistently across modeled outcomes, we regressed Year; county-level child Overweight/Obese populations, schools’ Academic Growth Status throughout the study period; School Income; Elementary School designation; School Locale; School Racial/Ethnic Demographics; and events, programs, and policies. The final four independent variables also served as dependent variables in this analysis. The team theorized, based on communications with ARTS Coordinators and in keeping with the theory of Diffusion of Innovations [30], that school communities deciding whether to adopt ARTS programming would likely start by experimenting with activities that require less effort. Then over time, assuming events are reviewed favorably by students, parents, and school personnel, schools would opt to institute more recurring and far-reaching walking and bicycling-promotive programs and policies. Table 2 displays the results of these two-level mixed effects logit models.

**Table 2:** Multilevel, mixed-effects logit model odds ratios depicting the relative odds of engaging in BTSD, WTSD, Programs, and Policies

*Predictor*	BTSD	WTSD	Program	Policy
*Year*				
2013 (referent)	–	–	–	–
2014	1.524 (0.460)	2.189** (0.342)	2.549 (2.871)	0.071 (0.802)
2015	3.220** (0.882)	2.477** (0.378)	13.887* (7.318)	19.127** (5.226)
2016	3.944** (1.069)	3.760** (0.554)	13.246* (6.868)	2.142* (0.288)
2017	5.902** (1.637)	3.506** (0.522)	7.162 (7.234)	23.054** (5.101)
*Overweight/Obese*	1.023 (0.409)	2.021** (0.478)	1.162 (0.455)	5.357 (4.789)
*Academic Growth Status*				
Exceeded (referent)	–	–	–	–
Met	0.948 (0.168)	1.05 (0.120)	1.328 (0.410)	0.924 (0.422)
Not Met	0.920 (0.199)	0.858 (0.121)	0.862 (0.402)	0.712 (0.315)
*School Income*				
High (referent)	–	–	–	–
Medium	0.603* (0.134)	0.677 (0.106)	1.963 (0.969)	0.711 (0.431)
Low	0.739 (0.258)	0.775 (0.174)	4.752* (2.813)	0.503 (0.402)
*Elementary School*	3.914** (1.151)	3.042** (0.465)	0.567 (0.245)	0.936 (0.414)
*School Locale*				
City (referent)	–	–	–	–
Suburb	1.427 (0.329)	0.735* (0.109)	2.424 (1.188)	1.398 (0.311)
Town	1.490 (0.527)	0.622* (0.136)	2.214 (1.104)	0.859 (0.314)
Rural	1.184 (0.274)	0.555** (0.081)	2.128 (1.158)	0.730 (0.185)
*School Racial/Ethnic Demographics*				
Hispanic	0.988 (0.274)	0.519* (0.117)	0.632 (0.484)	1.092 (0.397)
Black	0.825 (0.239)	0.738 (0.127)	0.297 (0.211)	1.178 (0.330)
White	0.994 (0.171)	0.718 (0.121)	0.929 (0.256)	0.686 (0.168)
Two Races	–	0.551 (0.221)	–	0.779 (0.510)
*BTSD*	–	16.163** (2.686)	3.077** (0.832)	2.010 (1.299)
*WTSD*	16.483** (2.725)	–	17.513** (4.824)	6.095** (2.570)
*Program*	3.509** (0.934)	16.127** (3.073)	–	2.333 (1.267)
*Policy*	1.535 (1.112)	2.741** (0.449)	1.495 (0.681)	–
Constant	0.001** (0.000)	0.009** (0.002)	0.000** (0.000)	0.000** (0.000)
Observations	13,010	13,010	13,010	13,010
Log likelihood	− 1264.475	− 2685.341	− 471.879	− 723.022
X^2^	819.360	938.260	234.990	209.490
*p*	0.000	0.000	0.000	0.000

*Note.* Standard errors in parentheses; Grouping variable = NC county. *p < .05. **p < .01.

*BTSD* Bike to School Day; *WTSD* Walk to School Day; Program = bicycle or pedestrian safety education or promotional efforts that recur at least once per month; Policy = policies to incorporate bicycle or pedestrian education of programming into school procedures or curricula

Following our estimation of two-level mixed effect logit models in this analysis, the team calculated intraclass correlation coefficients for each of the models we fit. Table 3 displays these intraclass correlation coefficients, which indicate the degree of similarity among schools from the same county on the schools’ likelihood to participate in Project outcomes of interest.

**Table 3:** Intra-class correlations among schools within NC counties by outcome

Outcome	ICC	SE
BTSD	0.271	0.065
WTSD	0.235	0.042
Program	0.451	0.102
Policy	0.873	0.073

*Note. ICC* Intraclass correlation coefficient

*SE* Standard error of the estimate

## Results

As seen in Fig. 1, an increasing proportion of NC schools engaged in ARTS programming from 2013 through 2017. For example, their engagement with “BTSD” rose over time, especially starting in 2015. On the other hand, schools’ involvement with “WTSD” events increased steadily from 2013 through 2017. A modest percentage of schools established recurring “Programs” in 2015, with two dozen more schools establishing Programs in 2017. Also beginning in 2015, more and more districts and schools enacted walking and bicycling-promotive “Policies.” “Any Participation” in the ARTS Project—a summation of the four Project outcomes of interest—increased substantially, reaching a “critical mass” of ARTS Project participation between 2015 and 2016, i.e., between the second and third year of Project rollout.

The following narrative of study findings corresponds with the multilevel, mixed-effects logit model results displayed in Table 2.

### Bike to school day (BTSD) and walk school day (WTSD) participation

Compared to the year before the start of the Active Routes to School—2013—NC schools’ participation in Bike to School Day (BTSD) increased significantly. By 2017, schools were nearly six times more likely than in 2013 to register a BTSD event. A similar trend emerged with Walk to School Day (WTSD) registrations among NC schools. Compared with 2013, in recent years—2016 and 2017, schools were between three and four times more likely to register WTSD events.

Registering a BTSD event was also predicted by elementary school—relative to middle school—status, with a four-time higher likelihood of registering a BTSD event. Medium-income schools were only 60% as likely to register for BTSD as low-income and high-income schools. Registering a WTSD event strongly predicted schools’ registration of BTSD events, being 16 times more likely to register for a BTSD event if the schools registered WTSD events, and vice versa. Schools’ locale did not significantly vary across categories when it came to registering BTSD events, however, the odds of schools located in cities registering WTSD events was 27% higher than schools in suburbs, 45% higher than schools in towns, and 1.8 times higher than schools in rural areas. Schools with bicycle or pedestrian promotive policies were 2.7 times more likely than schools without such policies to register WTSD events.

### Establishment of recurring bicycle or pedestrian education or promotional programs

Relative to baseline (2013) and the first complete year of the ARTS project (2014), in 2015 and 2016, NC schools were significantly more likely to engage in recurring educational or promotional programs for walking or bicycling to or at school. Low income schools were 4.8 times as likely as their high-income peers to participate in recurring walking or bicycling programs. And schools that had registered BTSD or WTSD events were 3 and 17.5 times more likely to engage in recurring programs, respectively, than schools that had not participated in these promotional events.

### Institution of bicycle or pedestrian promotive policies

The multi-level mixed effects logit models used in this analysis indicate that school- and district-level policies were significantly more likely to be instituted in 2015 and 2017, and to a lesser extent in 2016, than policies appeared at baseline (2013) and the first year of the project (2014). Schools’ and districts across geographic locale categories (i.e., city, suburb, town, rural) were equally likely to institute walking and bicycling-facilitative policies. Further, schools that participated in WTSD events were 6.1 times more likely than non-participating schools to enact facilitative policies.

### Variance in schools’ participation in project outcomes attributable to county-level clustering

The intraclass correlations from Table 3 suggest that schools from the same county—and school district for most public schools in NC—were more similar to each other than they were to schools from different counties. County-clustered schools’ similarity is demonstrated in the amount of variation in modeled outcomes that can be explained by the clustering of schools into their respective counties. For example, about 27% of the variation among schools’ participation in BTSD events can be explained by the county in which the schools are located. However, there was increasingly less within-county variation among schools that participated in recurring walking or bicycling programs and especially among schools that instituted pro-walking and bicycling policies. Much of the higher within-county correlations for programs and policies is probably attributable to the fact that many of these outcomes are implemented across all schools in a county, such as recurring bicycle education programs whereby all teachers in a school district (typically determined by counties in NC) received training on how to conduct on-bicycle safety education. In the case of walking- and bicycling-facilitative policies, many of these were implemented at the school district level and thus applied to all schools within the district (i.e., county).

## Discussion

In this study, the research team explored the uptake and reach of the Active Routes to School (ARTS) Project among K-8th grade schools in North Carolina (NC). Investigating schools’ participation in the Project from 2013 through 2017 revealed that their engagement with ARTS programming increased markedly over this time frame. Regarding our first research question about the extent to which disadvantaged rural schools participated in the ARTS Project, results indicate that lower income schools were more likely to establish recurring bike and walk programs than their wealthier counterparts. Further, schools located in rural areas were less likely than city schools to participate in WTSD or to institute bike- and walk-promotive policies. However, rural schools were as likely as schools in larger, denser locations to participate in BTSD and to establish recurring bike and walk programs.

Regarding our second research question—which pertained to ways in which schools’ participation in the ARTS Project varied according to schools’ rurality and socio-demographics—lower income and rural schools in NC tended to favor bike- and walk-facilitative programs over events (i.e., WTSD or BTSD) and policies. This orientation toward recurring programs may be partially explained by the social significance often assigned to rural school institutions. As prior work has demonstrated, schools in rural locales in the US and NC often serve as civic centerpieces in their regions, providing educational, recreational, and political services to rural communities [31, 32]. In rural locations without supportive biking and walking infrastructure and municipalities’ inability to finance skills-based programming, families living in these areas often depend on local schools to provide them with opportunities to acquire biking and pedestrian safety skills and regular physical activity [33].

Across all NC schools, participation was especially common in Walk and Bike to School Day (WTSD and BTSD) events. Participation in these events—especially WTSD—appears to have predicted engagement with recurring biking and walking programs, and with instituting biking- and walking-facilitative policies. This finding is consistent with prior work that has illustrated the potential of promotional events to inspire engagement in more recurring programming [34].

Results also suggest that the ARTS Project aligned with principles of Diffusion of Innovations. This theory explains how innovations spread across members of social systems over time [30]. The structure of the ARTS Project possesses several of the principles that Dearing outlines in his work on “designing for diffusion” [35]. That is, to design an innovation for diffusion throughout a social system, intervention teams should set up programs to ensure that the innovation is noticed by potential adopters, positively perceived by them, experimented with, and discussed among members of the social system.

Considering a few key attributes of the ARTS Project interventions illustrate how the interventions effectively diffused across schools and school districts in NC. Drawing upon the synthesis that Rogers [30] developed, one can see how ARTS programming adhered to characteristics of innovations that diffuse through social systems (Table 4).

**Table 4:** Illustrations of how the ARTS project adhered to characteristics of innovations that diffuse through social systems (adapted from Rogers, 2003)

Characteristics of Innovations that Diffuse	Illustrative attributes of the ARTS Project
Relative advantage	Compared to other physical activity programs, engagement with the ARTS Project tended to require less space, equipment, and class time to carry out or implement.
Trialability	One-time promotional events (e.g., BTSD and WTSD) typically did not require ongoing financial or administrative commitments, yet often led to greater involvement with the Project.
Compatibility	The ARTS Project and its promotion of physical activity was in keeping with many schools’ and districts’ goals to foster health and well-being among their students and staff.
Observability	The benefits of participating in the ARTS Project were often salient to parent groups, and school staff and administrators in the form of more alert, happier, and better performing students.
Simplicity	Acting as change agents in the diffusion of the ARTS Project, ARTS Coordinators illustrated the ease of establishing recurring practices including the “morning mile” and promotional events (e.g., WTSD and BTSD) toward building up more comprehensive active school travel programs.

In addition to the attributes of the ARTS Project itself are the structure of the social system of potential innovation adopters and the actors within the system. Dearing illustrates how designers of interventions can draw upon validated concepts from Diffusion of Innovations literature to accelerate the rate of the interventions’ adoption [35]. Among the concepts Dearing reviews, several seem to apply to the ARTS Project experience: “intervention clusters”; “intervention adaptation”; “demonstration projects”; and “societal sectors.”

The ARTS Coordinators worked with schools and districts in NC to group logical interventions together into “intervention clusters”—or packages of complementary interventions—toward making project elements more appealing and consistent with schools’ and districts’ physical and social realities. For example, given their relative remoteness from students’ residences, rural school administrators were often attracted to the educational aspects of the ARTS Project (e.g., pedestrian safety training, and on-bike stills training) rather than the promotional interventions designed to motivate students to walk or bike between home and school. In this way, the ARTS Coordinators also worked with project-adopting schools to partake in “intervention adaptation”, which can enhance adopters’ ownership of interventions and increase the interventions’ chances of being sustained in practice [36].

Furthermore, ARTS Coordinators frequently shared inspirational narratives about projects that other schools and districts within their regions had implemented. Descriptive narratives can help illustrate the utility of the innovation to potential adopters [37] and thus heighten future adopters’ interest in the experimenting with the interventions.

Toward accelerating the adoption of ARTS project interventions, the ARTS Coordinators often conceived of schools and their districts as “societal sectors”, which represent groups of similar organizations with similar missions. As discussed in the literature on the Diffusion of Innovations, tapping into professional networks that exist among members of societal sectors can greatly expand the reach of interventions [35, 37]. The ARTS Project’s focus on schools and districts as adopting entities likely contributed to the Project’s rapid diffusion through these societal sectors across diverse and even remote parts of NC.

Regarding the role of the ARTS Coordinators, these professionals served as “change agents”, linking a “resource system” with a “user system” [38]. That is, they connected state-provided resources in the form of trainings, safety materials, and structured promotional programs with schools and districts desiring to reduce school campus traffic congestion, enhance academic performance, and improve student health.

The ARTS Project shows potential for engaging harder to reach communities in learning bicycle and pedestrian safety skills and partaking in regular physical activity. Throughout the evolution of the Project it has become clear that the ARTS Coordinators engaged with an increasingly diverse set of partners. Such a “collaborative action” model, made possible through a high degree of coordination among community organizations and public health practitioners, can ameliorate health inequities by integrating and compounding models of support for traditionally marginalized populations [39].

### Limitations

Though this research reveals encouraging findings about the reach of the ARTS Project in NC, this study possesses several noteworthy limitations. For one, over the course of the project, the team revised its operationalization of intervention activities. For example, an activity designated as a “pedestrian safety training” may have involved classroom instruction on safe ways of crossing the street, or experiential education and practice crossing real roadways under the supervision of adults. This is an important distinction, as a review by Schwebel and colleagues suggests that only trainings designed to target specific unsafe pedestrian behaviors (e.g., dash-out prevention) and use real-life or life-like settings are effective at improving child pedestrian safety [40].

Another study limitation included the research team’s inability to estimate the number of students who had participated in ARTS programming. In the case of district-level policies, one could argue that such policies likely impacted all students within the district. Conversely, it was difficult to estimate the number of students who engaged with various Project outcomes per school each year. For example, in the case of recurring biking and walking programs, student participation varied on weekly and monthly bases, and frequently within the same school. The shifting nature of student participation in the Project rendered yearly estimates unreliable. Additionally, the ARTS project was financed using “non-infrastructure” funding from the US federal program, SAFETEA-LU. Though NCDOT implemented SAFETEA-LU-funded infrastructure projects throughout the study period, the research team lacked access to the timing and placement of such infrastructure. Not knowing where concurrent bicycle and pedestrian infrastructure was installed to complement ARTS programming is a significant shortcoming of this study.

Furthermore, though we estimated the amount of variability in schools’ adoption of ARTS Project interventions attributable to the schools’ district (county), the research team did not explore those county/district-level policies, procedures, communications, etc. that may have facilitated or suppressed participation in the ARTS Project. For example, did any of the districts ban walking to biking to school? We also failed to capture school-level “cultural” factors that likely contribute to intervention adoption. It is possible that intervention schools in this study were a self-selected group, which may bias results to favor those schools “ready” or otherwise “motivated” to promote active school travel or bicycle and pedestrian safety (e.g., schools that during the study period were looking to address childhood obesity or meaningfully respond to a recent crash involving a child pedestrian). Lastly, considering the scope of this study, we are unable to generalize study results outside of the state of North Carolina.

### Ideas for future research

The present study examines initial stages of engagement with the statewide NC ARTS Project. Researchers could extend this study by examining additional collaborative governance arrangements [41] formed in other US states and how such arrangements explain and predict students’ engagement with biking and walking to school. One purpose of this future research would be to uncover arrangements that afford more robust and socially inclusive action around safe and active school travel. A complementary line of inquiry could involve observing the extent to and conditions under which program adoption leads to implementation, institutionalization, and finally, to desirable outcomes (e.g., more students biking and walking between home and school; improvements in cyclist and pedestrian safety among intervention schools and neighborhoods). For example, how do schools institutionalize bicycle safety skills training into their curricula? Alternatively, under what conditions does the installation of biking- and walking-supportive infrastructure alter the ways in which families and school staff arrange their school travel patterns? These and many other questions could serve to help funders, practitioners, and stakeholders develop and operate more effective programs.

## Conclusion

The research team explored the adoption of the Active Routes to School (ARTS) Project among K-8th grade schools in North Carolina. From 2013 through 2017, schools’ and districts’ participation in the ARTS Project increased significantly. Much of this participation involved low commitment activities such as organizing a Walk to School Day event yet experimenting with walking or biking to school in this way often predicted schools’ establishment of recurring biking and walking programs and walking and biking-facilitative policies. Moreover, mixed effects logit models demonstrated that meaningful participation in the ARTS project occurred regardless of schools’ income, racial or ethnic makeup, or rurality. With a critical mass of schools now instituting continuous walking and biking programs and supportive policies, ARTS Project interventions are on a trajectory to positively impact growing numbers of school communities.

## Data Availability

The study data are available upon request to the corresponding author.

## Abbreviations

ARTS: Active Routes to School
BTSD: Bike to School Day
DOT: Department of Transportation
DPH: Division of Public Health
EVAAS: Education Value-Added Assessment System
MPO: Metropolitan Planning Organization
NC: North Carolina
NCES: National Center for Education Statistics
SAFETEALU: Safe, Accountable, Flexible, Efficient Transportation Equity Act, A Legacy for Users
SRTS: Safe Routes to School
WTSD: Walk to School Day
